# Neural regulation of tumor progression: implications for hepatocellular carcinoma

**DOI:** 10.3389/fimmu.2026.1884297

**Published:** 2026-06-19

**Authors:** Yalin Chen, Zhanghui Long, Yuan-Sheng Zang, Dong Wu

**Affiliations:** 1 The First Department of Hepatic Surgery, Eastern Hepatobiliary Surgery Hospital, Naval Medical University, Shanghai, China; 2 Department of Medical Oncology, Shanghai Changzheng Hospital, Naval Medical University, Shanghai, China

**Keywords:** hepatocellular carcinoma, immunosuppression, neuro-tumor interactions, perineural invasion, tumor microenvironment

## Abstract

The nervous system is increasingly recognized as an active regulator of oncogenesis rather than a passive structural component of the tumor microenvironment (TME). Neural activity promotes tumor proliferation, invasion, metastasis, therapeutic resistance and immune evasion through dynamic, bidirectional interactions with malignant cells and stromal components. These effects are mediated by diverse mechanisms, including synaptic and pseudo-synaptic communication, paracrine release of neurotransmitters and neuropeptides, metabolic reprogramming, and neuro-immune crosstalk. Importantly, tumors do not simply receive neural signals; they actively remodel the peripheral nervous system by inducing axonogenesis and neo-innervation, thereby establishing feed-forward circuits that facilitate disease progression. In hepatocellular carcinoma (HCC), central nervous system responses to systemic stress converge with local hepatic signaling to regulate tumor dynamics. Sympathetic, parasympathetic, and sensory innervation influence hepatocarcinogenesis by modulating inflammation, immunosuppression, stromal remodeling and cellular metabolism. These processes may contribute to the transition from chronic liver disease to malignant transformation. Although recent studies have demonstrated neuro-tumor crosstalk in HCC, critical knowledge gaps remain, including the precise neural circuits involved, the temporal dynamics of innervation during disease progression, and the intersection of these pathways with canonical oncogenic drivers. This review summarizes current knowledge of neural-mediated tumor progression across malignancies and discusses the relevance of these paradigms to HCC, with an emphasis on potential therapeutic vulnerabilities. A deeper mechanistic understanding of these processes is essential for translating neuro-oncological concepts into targeted neuromodulatory therapies for HCC.^1^

## Introduction

The nervous system regulates the development, plasticity, homeostasis and regeneration of non-neural tissues, raising the possibility that analogous mechanisms may contribute to cancer initiation and progression (1, 2). Over the past decade, this concept has challenged the traditional view that nerves are passive structures merely infiltrated by tumors. Accumulating evidence now supports an active role for the nervous system in tumor biology through complex and dynamically regulated networks (3). Neurons release neurotransmitters, neuropeptides, and other signaling molecules into the tumor microenvironment (TME), where these factors engage specific receptors on malignant cells and promote proliferation, migration, invasion and survival (4, 5). These neural signals also intersect with canonical oncogenic cascades, including the MAPK, PI3K/AKT and cAMP-PKA pathways, thereby amplifying tumor-promoting programs (6–8). Conversely, tumors are not passive recipients of neural input. Malignant cells and stromal components secrete inflammatory mediators, chemokines and neurotrophic factors that induce neural remodeling, enhance neuronal excitability, and reinforce bidirectional feed-forward signaling (9–11). The immune system further serves as a critical intermediary in neuro-tumor crosstalk, as neural signals modulate the recruitment and function of immune cells and shape a microenvironment favorable to tumor growth (12–14). Despite growing interest in this field, several issues remain unresolved, including the heterogeneity of nerve-tumor interactions across malignancies and the molecular mechanisms that govern these signaling networks. This review synthesizes emerging insights into the neural regulation of hepatocarcinogenesis and hepatocellular carcinoma (HCC) progression.

## The nervous system: organization and functional features relevant to cancer

Elucidating the neural mechanisms underlying oncogenesis requires an understanding of neural architecture and its regulatory influence on the TME. Far from being a passive bystander within the tissue, the nervous system functions as a dynamic, spatiotemporally regulated network. It modulates cellular behavior, maintains tissue homeostasis, and orchestrates systemic responses under both physiological and pathological conditions (15).

Structurally, the nervous system is broadly divided into two major components: the central nervous system (CNS) and the peripheral nervous system (PNS). The CNS, comprising the brain and spinal cord, serves as the principal integrative center for emotion, cognition, and behavior. By regulating stress responses, circadian rhythms and neuroendocrine output, the CNS converts psychological and environmental inputs into sustained physiological signals. The PNS extends beyond the CNS and distributes neural impulses throughout the body, linking central regulatory programs to peripheral tissues and organs (16–18). These centrally derived signals are relayed primarily through the hypothalamic-pituitary-adrenal (HPA) axis and autonomic efferent circuits, thereby exerting systemic effects on peripheral organs such as the liver (19, 20). Within the PNS, the autonomic nervous system comprises sympathetic and parasympathetic divisions that regulate visceral functions, including circulation, metabolism, and immune activity. In parallel, sensory nerve fibers, including nociceptors, transmit peripheral information to the CNS and locally release neuropeptides that modulate inflammation and cellular behavior (21–23). Thus, neural components do not operate in isolation but form integrated, reciprocal networks that adapt dynamically to tissue injury, chronic inflammation, and disease progression.

Neural regulation of peripheral tissues is mediated through three major signaling modalities. First, electrical propagation enables rapid and high-fidelity transmission along axons, coordinating spatially distributed physiological responses. Second, chemical signaling occurs through the release of neurotransmitters, such as catecholamines and acetylcholine (ACh), as well as sensory neuropeptides. These molecules bind specific receptors on non-neural cells and transduce neural activity into downstream intracellular signaling cascades. Third, the nervous system forms integrated networks with the endocrine and immune systems. These neuroendocrine-immune axes coordinate systemic stress adaptation, modulate inflammatory responses, and maintain metabolic homeostasis (24).

This structural and functional organization endows the nervous system with the capacity to regulate critical biological processes, including localized inflammation, immune surveillance, tissue remodeling, and cellular phenotypic plasticity. However, neural activity is rarely the singular, initiating driver of tumorigenesis. Rather than acting as direct oncogenic effectors, neural signals function as contextual modulators. They exert their influence through dynamic, context-dependent interactions with established oncogenic mutations and various components of the TME. Recognizing this mechanistic nuance is critical for delineating how neuromodulatory pathways facilitate both tumor initiation and subsequent malignant progression.

## Experimental strategies for neural blockade and modulation

Given the emerging role of neural signaling in tumor progression, interrogation of these pathways has become an important research priority. Current experimental strategies for investigating neuro-tumor crosstalk generally aim to suppress neuronal activity, disrupt neurotransmission, or manipulate defined neuronal populations. These approaches include clinical observations, *in vivo* animal models, and *in vitro* cell-based systems. Each method captures a distinct level of neural regulation and collectively provides mechanistic insight into how neural inputs modulate tumor biology.

### Clinical evidence

In clinical settings, neural signaling is often incidentally modulated by medications prescribed for non-oncological indications. For example, β-adrenergic receptor antagonists (beta-blockers) used for cardiovascular disease and anticholinergic agents prescribed for gastrointestinal or neurological disorders can attenuate autonomic neurotransmission. Retrospective epidemiological studies have used these pharmacological exposures to evaluate cancer incidence, progression, and clinical outcomes in patients receiving long-term autonomic modulation, including patients with HCC (25, 26). In addition, iatrogenic nerve injury during surgical procedures may cause localized denervation. Although these clinical scenarios are observational and do not provide controlled neuromodulation, they offer preliminary evidence that chronic disruption of neural signaling can influence tumor behavior.

### Animal models

Animal models allow direct interrogation of neural function *in vivo*. Chemical denervation is frequently used; for example, the neurotoxin 6-hydroxydopamine (6-OHDA) selectively ablates sympathetic nerve fibers (27). This approach enables investigation of how reduced adrenergic signaling alters tumor growth, local inflammation, and immune infiltration (28, 29). Surgical denervation provides a physical method for interrupting organ-specific nerve supply, although it is technically demanding and may induce compensatory neural rewiring. More recently, neuron-specific genetic tools have advanced the field substantially. Conditional gene ablation, chemogenetics, and optogenetics provide precise spatiotemporal control over defined neuronal populations (30–34), enabling dissection of the contributions of specific neural circuits to tumor progression in living hosts.

### Cell-based systems

Reductionist *in vitro* systems provide a useful platform for mechanistic studies. By exposing tumor, immune or stromal cells to exogenous neurotransmitters or neuropeptides, investigators can evaluate receptor-dependent signaling cascades. These pathways can also be modulated with specific pharmacological antagonists or by targeted genetic manipulation of neural receptors. Co-culture systems that combine neuronal or neuron-like cells with malignant cells or TME components provide a more refined model for studying bidirectional paracrine communication. Although these *in vitro* models do not recapitulate the systemic complexity of an intact organism, they are valuable for isolating individual cell types and defining the molecular interactions that govern neuro-tumor crosstalk.

Together, these approaches constitute the primary experimental framework for studying neural blockade and modulation in oncology. The integration of clinical observations, *in vivo* animal models, and *in vitro* cellular systems provides a comprehensive basis for elucidating how neural inputs shape tumor progression, immune regulation, and therapeutic responses, particularly in HCC.

## Functional coupling between nerves and tumors

Having outlined the structural basis of neural regulation and the experimental strategies used to interrogate it, we next examine how neural inputs shape tumor phenotypes. Increasing evidence indicates that nerve-tumor interactions extend beyond diffuse paracrine signaling and may involve stable, dynamically regulated structural and functional coupling (35, 36). Through sustained neurotransmitter release, synapse-like communication, and receptor-dependent signal transduction, neural activity can promote tumor cell proliferation, invasion, and metastasis. It can also facilitate immune evasion by remodeling the tumor immune microenvironment. Collectively, these observations establish functional neuro-tumor coupling as an important mechanism of cancer progression.

### Neural activity promotes tumor cell proliferation

High-grade gliomas provide a well-characterized example of direct neural control of tumor proliferation. Single-cell transcriptomic and ultrastructural studies have shown that glioma cells express synapse-related programs and can receive excitatory neuronal input through α-amino-3-hydroxy-5-methyl-4-isoxazolepropionic acid (AMPA) receptor-mediated neuron-tumor synapses, leading to membrane depolarization and increased proliferation (37–39). Glioma growth is also influenced by soluble neuronal factors, including brain-derived neurotrophic factor (BDNF), glucose-regulated protein 78 (GRP78), and neuroligin-3 (NLGN3) (40–44). More recent evidence shows that neuromodulatory cholinergic circuits also contribute to glioma growth. In diffuse midline glioma, midbrain cholinergic neuronal activity promotes tumor proliferation through ACh-dependent activation of M1/M3 muscarinic receptors on tumor cells (45).

Direct exploitation of neural input is not restricted to CNS malignancies; peripheral tumors can also hijack host neural circuits through direct synaptic connections to acquire survival and growth signals. For example, small cell lung cancer (SCLC) cells form functional pseudo-synapses with peripheral neurons and receive neural input through N-methyl-D-aspartate (NMDA) and gamma-aminobutyric acid type A (GABA_A_) receptors, thereby promoting tumor expansion (46). Beyond direct synaptic communication, neurons may promote tumor progression through paracrine signaling. In triple-negative breast cancer (TNBC), which is densely innervated by sympathetic fibers, neuron-derived norepinephrine (NE) binds tumor-expressed β-adrenergic receptors (ADRB2), accelerating tumor cell proliferation and nerve growth factor (NGF) secretion (47). Similarly, in pancreatic ductal adenocarcinoma (PDAC), sympathetic nerve-derived NE activates ADRB2 and induces NGF and BDNF secretion, which stimulates sympathetic fiber hyperplasia, increases tumor innervation, and accelerates disease progression (48). In the gastric mucosa, doublecortin-like kinase 1 (DCLK1)-positive tuft cells and local nerve fibers serve as major sources of ACh. ACh activates gastric epithelial cells to upregulate NGF, promote enteric nerve fiber expansion, stimulate aberrant epithelial proliferation, and ultimately facilitate tumorigenesis (11).

Together, these studies indicate that neural inputs sustain oncogenic proliferative signaling through two major mechanisms: direct synaptic communication mediated by neurotransmitter release and paracrine secretion of neurotrophic factors. Both modalities can contribute to tumor initiation and sustained expansion.

### Neural activity promotes tumor invasion and metastasis

In glioblastoma (GBM) and brain metastasis, neuronal activity may facilitate invasion and colonization by coupling tumor cells to local neural circuits. In GBM, invasion-associated tumor subpopulations can acquire neuron-like and neural progenitor-like features, receive glutamatergic inputs, and use AMPA receptor-dependent calcium signaling to promote tumor microtube formation and intracerebral dissemination (49). Related work indicates that invasive GBM cells may form neuron-tumor networks and migrate along axonal tracts, while radiotherapy-induced neuronal activity can strengthen such connectivity and contribute to therapeutic resistance (50). Similar spatial proximity to excitatory synapses enables metastatic tumor cells in the brain to activate NMDA receptor signaling and support outgrowth (51). These CNS studies provide a structural and functional framework for neuro-tumor coupling, although their direct relevance to HCC remains extrapolative.

In contrast to CNS tumors, the pro-invasive and pro-metastatic effects of neural activity in peripheral malignancies predominantly involve paracrine signaling and remodeling of the nerve-microenvironment interface. Magnon and colleagues demonstrated that the autonomic nervous system exerts dual regulatory effects in prostate cancer: sympathetic nerves promote early tumor growth through β2/β3-adrenergic receptors, whereas parasympathetic cholinergic fibers promote invasion and metastasis through muscarinic receptors, particularly the M1 subtype (10). In breast cancer, tumor cells induce spontaneous calcium activity in sensory neurons, triggering release of the neuropeptide substance P (SP). SP engages tachykinin receptor 1 (TACR1) on tumor cells and induces targeted apoptosis in a small subset of TACR1-high cells. These dying cells release single-stranded RNA into the TME, activating Toll-like receptor 7 (TLR7) in neighboring tumor cells and initiating atypical prometastatic transcriptional programs (52). In PDAC, neuron-derived glutamate induces calcium influx through NMDA receptors, activates the calcium/calmodulin-dependent protein kinase II (CaMKII)/ERK-MAPK pathway, and upregulates methyltransferase-like 3 (METTL3). METTL3 subsequently increases hexokinase 2 (HK2) expression through N6-methyladenosine (m6A) modification, accelerating glycolysis and facilitating perineural invasion (PNI) (53).

Traditionally, PNI was regarded as a path of least mechanical resistance for cancer cell dissemination. Emerging evidence, however, indicates that bidirectional interactions between nerves and malignant cells actively coordinate this process. Under physiological conditions, Schwann cells primarily maintain peripheral nerve integrity. In the pancreatic TME, however, Schwann cells are reprogrammed to form dynamic structures termed tumor-activated Schwann cell tracks (TASTs), which facilitate cancer cell invasion (54). Cancer cells induce this transformation, prompting Schwann cells to adopt a non-myelinating, repair-like phenotype that organizes invasive tracks and mechanically supports cancer cell motility. These observations highlight the active role of Schwann cell transdifferentiation in metastatic dissemination.

### Neural activity shapes an immunosuppressive tumor microenvironment

Immunoregulation is a central dimension of neuro-tumor crosstalk and is reflected in changes in T-cell differentiation, infiltration, and function. In melanoma, sympathetic nerves regulate tumor-infiltrating lymphocytes through ADRB2 on CD8^+^ T cells (55). Sympathetic-derived catecholamines also induce CD8^+^ T-cell exhaustion through ADRB1 signaling in both melanoma and PDAC (56). In orthotopic PDAC models, ACh suppresses CD8^+^ T-cell recruitment by downregulating tumor CCL5 expression and directly inhibiting interferon-gamma (IFN-γ) production, thereby shifting the local immune landscape toward an immunosuppressive phenotype (57).

Recent studies indicate that neuro-immune modulation extends beyond lymphoid lineages to include myeloid populations. In GBM, neural activity promotes tumor growth and invasion, while the tumor reciprocally alters neural circuits to induce localized immunosuppression. Hyperconnected neuronal regions within tumors correlate with marked suppression of local inflammatory responses and altered infiltration and polarization of tumor-associated macrophages (TAMs) (58). In preclinical models, deletion of thrombospondin-1 (TSP1) in GBM cells disrupts tumor-neuron synaptogenesis and induces glutamatergic neuronal hyperactivity. This disruption upregulates genes related to antigen presentation, promotes infiltration of pro-inflammatory TAMs and CD8^+^ T cells, and alleviates TAM-mediated T-cell suppression. Similarly, pharmacological inhibition of glutamatergic signaling shifts TAMs toward a non-immunosuppressive phenotype, prolongs murine survival, and enhances the efficacy of cellular immunotherapies.

The neuro-immune dialog within PDAC also depends strongly on stromal intermediaries. Cancer-associated fibroblasts (CAFs) secrete NGF, which stimulates local nociceptors to release calcitonin gene-related peptide (CGRP). CGRP subsequently binds receptor activity-modifying protein 1 (RAMP1) on CAFs and inhibits interleukin-15 (IL-15) expression. This downregulation restricts natural killer (NK) cells infiltration and cytotoxicity, thereby accelerating PDAC progression and exacerbating cancer-associated pain (59). Similarly, in melanoma models, nociceptor neurons attenuate antitumor immunity by inducing CD8^+^ T-cell exhaustion (60).

Collectively, these findings indicate that neural activity does not regulate tumor cells in isolation. Instead, neurotransmitters and neural circuits remodel the broader TME, shaping the recruitment, polarization, and function of immune infiltrates and establishing a niche permissive to tumor progression.

**Figure 1:**
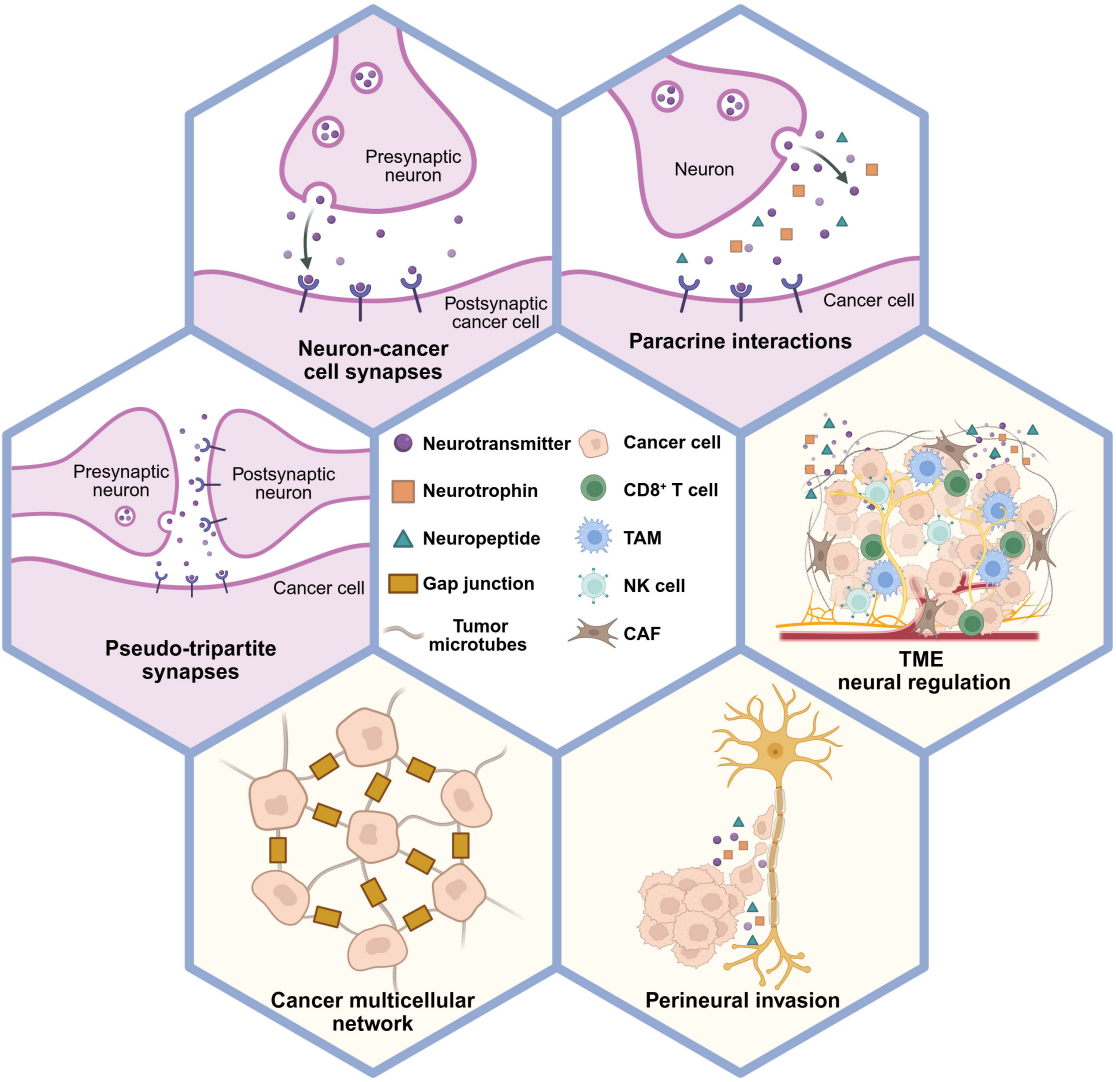
Mechanisms of neuro-tumor interactions.

As summarized in **Table 1**, **Figure 1**, these examples further support the emerging view that tumor innervation constitutes an important protumorigenic component of the tumor microenvironment.

**Table 1:** Neural mechanisms of tumor progression.

Functional effects	Tumor types	Neural types	Receptors	Mechanisms	References
Tumor cell proliferation	Glioma	Glutamatergic	AMPA	Neuron-tumor synapse	(38)
		Cholinergic	CHRM1 /CHRM3	Paracrine	(45)
	Lung	Glutamatergic	NMDA /AMPA	Neuron-tumor synapse	(46)
	Breast	Sympathetic	ADRB2	Paracrine	(47)
	Pancreatic	Sympathetic	ADRB2	Paracrine	(48)
	Gastric	Cholinergic	CHRM3	Paracrine	(11)
Tumor invasion and metastasis	Glioma	Glutamatergic	AMPA	TMs; Neuron-tumor synapse	(49, 50)
	Breast	Glutamatergic	NMDA	Pseudo-tripartite synapse	(51)
		Sensory	SP-TACR1	Paracrine	(52)
	Prostate	Cholinergic	CHRM1	Paracrine	(10)
	Pancreatic	Glutamatergic	NMDAR2B	Paracrine; PNI	(53)
				TASTs	(54)
Immunosuppressive TME	Glioma	Glutamatergic	AMPA	TME remodeling	(58)
	Melanoma	Sympathetic	ADRB1 /ADRB2		(55, 56)
		Sensory	CGRP-RAMP1		(60)
	Pancreatic	Sympathetic	ADRB1		(56)
		Cholinergic	nAChRs		(57)
		Sensory	CGRP-RAMP1		(59)

Neural activity may regulate tumor progression through direct neuron–tumor synapses, pseudo-synaptic contacts, and paracrine release of neurotransmitters, neuropeptides, and neurotrophic factors. These signals act on malignant, stromal, immune, and endothelial compartments and alter extracellular matrix organization within the TME. Perineural invasion represents a structural nerve–tumor interface involving axons, Schwann cells, matrix components, immune cells, and invading cancer cells. Their relevance to HCC depends on the strength of direct evidence.

## The nervous system in hepatocellular carcinoma

HCC is one of the most prevalent and lethal malignancies worldwide and is characterized by aggressive invasiveness, frequent recurrence, and resistance to systemic therapies (61–63). Historically, HCC research has focused primarily on tumor-cell-intrinsic abnormalities, including genetic mutations, epigenetic remodeling, and metabolic dysregulation, as well as the regulatory role of the immune microenvironment (64–67). Emerging basic and clinical evidence, however, highlights the nervous system as a regulatory network that integrates central control, peripheral stress responses, and local tissue homeostasis. This network exerts systemic and multilayered effects on HCC initiation, progression, and therapeutic response (68, 69). Neuro-oncological regulation is therefore increasingly recognized as an important component of liver cancer biology and provides a framework for understanding dynamic nerve-tumor interactions in HCC.

To avoid overinterpretation, the evidence discussed in the following sections is categorized according to its source and relevance to HCC. Findings derived from HCC tissues, HCC cell lines, or liver cancer animal models are regarded as HCC-specific evidence. Observations from chronic liver disease, fibrosis, cirrhosis, or general hepatic pathophysiology are described as liver-context evidence, as they provide a biologically relevant but indirect framework for hepatocarcinogenesis. Mechanisms established primarily in glioma, pancreatic cancer, breast cancer, prostate cancer, melanoma, or other malignancies are considered extrapolative evidence from other cancers and are discussed as conceptual parallels that require further validation in HCC.

### Anatomical and histological organization of hepatic innervation

During embryonic development, hepatic innervation undergoes progressive arborization, establishing a branched neural network composed of sympathetic, parasympathetic, and sensory fibers (70–72). These fibers converge at the hepatic plexus near the porta hepatis and course along the hepatic artery, portal vein, and biliary tree to enter the hepatic parenchyma. In the liver, nerve fibers are preferentially distributed around portal tracts, vascular structures, biliary compartments, fibrotic septa, and perinodular regions, forming spatially organized neurovascular and neuroimmune niches. Through this organization, neural signals may regulate hepatocytes, hepatic stellate cells (HSCs), Kupffer cells, endothelial cells, biliary epithelial cells, and immune infiltrates in a compartment-specific manner (73). Accordingly, the nerve–tumor interface in HCC may represent not only a neurotransmitter- or neuropeptide-mediated signaling axis, but also a microanatomical niche in which neural, vascular, stromal, immune, and malignant or premalignant epithelial components coexist. Although direct evidence in HCC remains limited, studies in other malignancies suggest that nerves can participate in tumor invasion and dissemination, as exemplified by perineural invasion (74).

### Central nervous system regulation in HCC

As the principal integration center for psychobehavioral inputs, CNS exerts organism-wide regulatory effects on disease progression and is increasingly recognized as an upstream modulator in cancer biology. Evidence linking CNS activity to HCC is currently strongest at the level of clinical association. Accumulating clinical and experimental evidence indicates that chronic psychological stress, depression, anxiety, and circadian disruption are associated with increased HCC risk and adverse clinical outcomes (75–79).

Mechanistically, the CNS transduces these psychobehavioral inputs into sustained neuroendocrine signals primarily through the HPA axis and the sympathetic nervous system (SNS), thereby altering the hepatic microenvironment (80, 81). Under chronic stress, sustained HPA-axis activation elevates systemic glucocorticoid levels, while persistent SNS hyperactivation induces prolonged release of catecholamines, including epinephrine (EPI) and NE. These neuroendocrine mediators affect hepatocytes and malignant cells directly and also remodel the hepatic immune architecture, thereby impairing systemic antitumor immunity (82).

At the immune microenvironment level, glucocorticoids inhibit dendritic cell (DC) maturation and antigen presentation while suppressing the cytotoxic activity of CD8^+^ T cells and NK cells (83, 84). Concurrently, catecholamines promote the expansion of immunosuppressive cell populations and activate protumorigenic inflammatory programs through adrenergic receptor signaling, establishing a niche conducive to tumor development and progression (82, 85).

As additional liver-context evidence, circadian disruption may exacerbate these effects by dysregulating the temporal coordination of central arousal pathways, including the HPA axis and SNS, and neuroendocrine secretion. This desynchronization disrupts hepatic metabolic homeostasis, lipid partitioning, and local immune surveillance, further accelerating hepatocarcinogenesis (86–90).

### Sympathetic regulation in HCC

Among the neural pathways discussed in this review, sympathetic regulation is supported by relatively strong HCC-specific evidence. EPI and NE are core mediators of sympathetic regulation in HCC. As tumors progress, β-adrenergic signaling becomes increasingly important. The β-adrenergic receptor ADRB2 is significantly upregulated in HCC cells and correlates with poor clinical outcomes. ADRB2 activation stimulates tumor cell proliferation, migration, and invasion. It also enhances cellular adaptation to hypoxia by inhibiting autophagic degradation and stabilizing hypoxia-inducible factor 1α (HIF-1α), thereby contributing to sorafenib resistance. Thus, adrenergic signaling may play an important role in therapeutic failure (91).

Consistent with this HCC-specific evidence, endogenous mechanisms within the TME can counteract adrenergic signaling. Monoamine oxidase A (MAOA) suppresses HCC invasion and metastasis by degrading EPI and NE, thereby attenuating ADRB2-epidermal growth factor receptor (EGFR) transactivation. This finding indicates that neurotransmitter catabolism can restrict malignant phenotypes (92). Chronic physiological or psychological stress, however, may disrupt this homeostatic balance. Under stress conditions, elevated EPI amplifies protumorigenic signaling through an ADRB2-ubiquitin-specific peptidase 10 (USP10)-pleomorphic adenoma gene-like 2 (PLAGL2) positive feedback loop, thereby promoting HCC progression (81).

At the level of inflammatory initiation, sympathetic nerves release NE to activate α1-adrenoceptors on Kupffer cells, triggering an inflammatory cascade that promotes hepatocarcinogenesis (93). In addition to their direct effects on tumor cells, EPI and NE remodel the immune and stromal landscapes of liver cancer. Under chronic stress, β-adrenergic signaling upregulates CXCL5, facilitating recruitment and intratumoral infiltration of myeloid-derived suppressor cells (MDSCs) through the CXCR2-ERK pathway and thereby exacerbating immunosuppression (94). Concurrently, NE activates HSCs to secrete secreted frizzled-related protein 1 (sFRP1), reinforcing a Wnt16B/β-catenin positive feedback loop that promotes HCC progression through stromal-tumor interactions (95). Furthermore, sympathetic signaling polarizes TAMs from an M1 phenotype toward a protumorigenic M2 phenotype, further compromising local antitumor immunity (96).

As liver-context evidence, the SNS remains chronically hyperactivated during prolonged liver diseases, including viral hepatitis, non-alcoholic steatohepatitis (NASH), and cirrhosis, leading to pathological accumulation of catecholaminergic neurotransmitters within the hepatic parenchyma (97–99). EPI, NE, and dopamine—the principal effectors of sympathetic signaling—exert distinct yet interconnected effects on HCC initiation, progression and therapeutic resistance through specific receptor subtypes and downstream signaling cascades.

Compared to EPI and NE, dopamine exhibits more complex, subtype-specific effects on HCC. Dopamine receptors DRD1 through DRD4 exert bidirectional regulatory effects across different cell types and pathological stages. DRD1 is primarily implicated in tumor suppression via the cAMP-PKA pathway, with elevated expression correlating with better differentiation and improved prognosis (100, 101). However, DRD2 activation has been shown to induce HSC activation and fibrogenesis under conditions of metabolic dysregulation and chronic inflammation, fostering a protumorigenic microenvironment (102, 103). In contrast, DRD3 functions predominantly as a tumor suppressor in HCC, and its high expression is associated with a favorable prognosis (104). The precise role of DRD4 remains poorly defined, though preliminary evidence suggests its involvement in cellular stress responses and proliferation (105, 106).

Collectively, sympathetic nerves may coordinate several HCC-related processes by integrating multiple catecholaminergic neurotransmitters and receptor subtypes. This axis is supported by relatively stronger HCC-specific evidence, including studies of ADRB2-dependent malignant phenotypes, sorafenib resistance, MAOA-mediated neurotransmitter catabolism, Kupffer cell-mediated inflammatory initiation, MDSC recruitment, HSC activation, and macrophage polarization. These findings indicate that sympathetic signaling represents a plausible therapeutic target in HCC biology, although clinical validation remains necessary.

### Parasympathetic regulation in HCC

The parasympathetic branch of the autonomic nervous system innervates the liver primarily through the vagus nerve, using ACh as its principal effector. ACh mediates its effects through both muscarinic and nicotinic cholinergic receptors. Emerging evidence suggests that parasympathetic cholinergic pathways not only maintain hepatic homeostasis but may also exert context-dependent tumor-promoting effects by coordinating tumor-cell-intrinsic signaling with changes in the immune microenvironment.

At the cellular level, HCC cells express diverse cholinergic receptors, enabling them to respond directly to ACh (107). ACh has been shown to activate the androgen receptor and non-canonically stimulate downstream pro-survival cascades, including the AKT and STAT3 pathways. These effects promote tumor cell migration and invasion while concurrently inhibiting apoptosis (108). Moreover, the α7 nicotinic acetylcholine receptor subunit (α7-nAChR) is closely associated with tumor cell survival and resistance to targeted therapy. Pharmacological inhibition of α7-nAChR markedly enhances the antitumor efficacy of sorafenib (109, 110).

At the level of immune regulation, vagus nerve-derived ACh has been reported to engage muscarinic receptors, particularly CHRM3, on CD8^+^ T cells, suppressing their effector functions and dampening systemic antitumor immunity (111, 112). *In vivo* hepatic vagotomy was reported to reduce liver tumor burden in an adaptive immunity-dependent manner, suggesting that vagal cholinergic signaling may contribute to immune evasion in liver cancer; however, part of this evidence remains preliminary and should be interpreted cautiously until further peer-reviewed validation is available (112). In addition, chronic cholinergic signaling may foster an inflammatory milieu during chronic liver disease, predisposing the microenvironment to malignant transformation (113).

Taken together, parasympathetic innervation establishes a cholinergic regulatory network that couples tumor-intrinsic pro-survival signaling with localized immunosuppression and thereby contributes to HCC progression. Compared with sympathetic regulation, however, the current evidence for parasympathetic regulation in HCC is more heterogeneous, comprising direct HCC-related evidence on cholinergic receptor signaling and more preliminary evidence regarding vagus nerve-mediated immune regulation.

### Sensory nerve regulation in HCC

Direct evidence in HCC for sensory nerve regulation remains limited. Therefore, this section mainly integrates liver-context evidence with extrapolative evidence from other solid tumors and should be interpreted cautiously. Sensory nerves, particularly nociceptor neurons, are important regulators of hepatic homeostasis and function as primary sensors of tissue injury, inflammation, and metabolic stress. In chronic liver disease, persistent inflammatory stimuli and tissue injury activate hepatic sensory nerve endings chronically, inducing localized release of neuropeptides such as CGRP and SP, which can remodel the TME (114, 115).

At the level of liver-context evidence, sensory nerves may influence HCC progression through interactions with the hepatic stroma. Sensory innervation in the liver can promote fibroblast activation and extracellular matrix remodeling. This crosstalk exacerbates hepatic fibrosis and chronic inflammation, contributing to the formation of a premalignant niche (60, 116). In immune regulation, CGRP binds its receptor complex to suppress pro-inflammatory mediator expression, attenuate the cytotoxic functions of NK cells and effector T cells, and establish an immunosuppressive microenvironment (117, 118). SP signals through neurokinin receptors, modulates inflammatory cascades, and facilitates context-dependent reprogramming of tumor-associated inflammatory responses (119, 120).

As extrapolative evidence from other solid tumors, analogous findings suggest that sensory nerves may actively participate in tumor initiation and progression through neuropeptide secretion and local immune modulation. Beyond immunomodulation, similar sensory nerve-mediated stromal and immune remodeling has been observed in other malignancies, supporting the biological plausibility of this mechanism in HCC, although direct validation remains necessary.

Furthermore, bidirectional communication between sensory afferents and the CNS may amplify these local effects at the systemic level. Persistent sensory activation exacerbates central stress responses, driving compensatory hyperactivation of sympathetic efferents and neuroendocrine axes. This systemic loop may intensify local immunosuppression and metabolic dysregulation within the liver (121, 122). Overall, current data suggest that sensory nerves exert a multilayered, neuropeptide-driven influence on local immunity and chronic inflammation. Modulating sensory-neural pathways may therefore represent a potential strategy for intercepting hepatocarcinogenesis in the context of chronic liver disease.

## Conclusions and future perspectives

Accumulating evidence supports a paradigm shift in cancer biology: neural regulation should no longer be regarded as a passive structural feature of the tissue stroma. Instead, the nervous system functions as an active component of the protumorigenic network. In both CNS and peripheral malignancies, neural activity modulates tumor behavior through direct synaptic communication, paracrine signaling, and metabolic or epigenetic reprogramming. These mechanisms contribute to tumor proliferation, invasion, metastasis, and therapeutic resistance.

In HCC, neural regulation integrates systemic stress responses with the local hepatic microenvironment. Sympathetic, parasympathetic, and sensory nerves may modulate hepatic inflammation, immunosuppression, stromal remodeling, and metabolic adaptation, linking chronic liver disease to malignant transformation and tumor progression. However, the evidence base remains uneven. Sympathetic signaling is supported by relatively stronger HCC-specific evidence, whereas cholinergic and sensory-neural mechanisms remain partly based on chronic liver disease models or extrapolative evidence from other tumor types.

Future studies should define the context- and stage-specific roles of distinct neuronal subpopulations, characterize the molecular determinants of tumor responsiveness to neural signals, and clarify how localized nerve–tumor crosstalk contributes to systemic dysfunction, including autonomic imbalance and circadian disruption. These efforts should be combined with spatial histology, neural manipulation models, and clinically annotated HCC cohorts.

Therapeutically, neural regulation offers potential opportunities but also important translational challenges. Adrenergic blockade, cholinergic modulation, and sensory neuropeptide targeting may represent candidate strategies, particularly in combination with immunotherapy, metabolic therapy, anti-fibrotic therapy, or standard systemic anticancer regimens. However, neuromodulatory interventions must be designed carefully to avoid disrupting cardiovascular regulation, metabolic homeostasis, pain signaling, or hepatic regeneration. A rigorous distinction between HCC-validated mechanisms and extrapolative concepts will be essential for developing precision neuromodulatory strategies for HCC.
